# Duplex Obturator

**Published:** 1894-09

**Authors:** J. W. Rhone

**Affiliations:** Bellefonte, Pa.


### International Dental Journal.

VOL. XV. SEPTEMBER, 1894. NO. 9.

### Original Communications.[1]

### DUPLEX OBTURATOR.[2]

BY DR. J. W. RHONE, BELLEFONTE, PA.

To enter upon a discussion of the anatomical construction of the normal palate and its functions in comparison with the abnormal, more particularly that of a congenital cleft palate, involving the hard and soft tissues, would seem unnecessary, as all seekers for information on these points can find them in our standard text-books, and can also compare the normal with the abnormal in the living.

Correcting all the evils of this malformation is more than should be expected, but the leading difficulties that can be remedied are defective articulation and enunciation, and the escape of solids and fluids through the nasal passages, and the difficulty of swallowing.

This brings us to the important point, How can these evils be best overcome?

Judging from the past history of the surgical side of this question, surgery has not proved a success in this line.

---

[1] The editor and publishers are not responsible for the views of authors of papers published in this department, nor for any claim to novelty, or otherwise, that may be made by them. No papers will be received for this department that have appeared in any other journal published in the country.

[2] Read before the Odontological Society of Pennsylvania by Dr. Rhone, and received from him but a few days before his death. It is probably the last contribution he made to the work of his profession.—ED.

It would then seem that mechanical appliances of some kind are the only means of relief at hand. But here we are again perplexed; the number and forms of these appliances are quite numerous, and all laying claim to something that seems new and novel. It, however, is not the purpose of this paper to attempt a discussion of the merits or demerits of any, excepting so far as may be necessary by way of comparison.

However, with comparatively limited experience in the construction and adaptation of appliances to be of benefit to patients of the class named, I would state that *they must be made in two parts* to bring about the best results and most comfort to the wearer.

The obturator in one piece *cannot be made* to compare in adaptation and comfort to the duplex. The reasons are plain; no part of the rigid (one piece) appliance can move without moving as a whole, which *will* produce irritation and discomfort at some points. With the duplex obturator, if properly constructed, this is avoided. The front half of the plate will be firm, while the posterior half, a hard rubber velum, attached to the plate by a hinge,[1] can move freely at any and every motion of the cleft uvula, and the wearer is enabled to talk or read aloud, and, not least, take food with comfort.

It might be in place here to state that about twenty-five years ago I made an obturator for a then young woman (who had a congenital cleft palate of both the hard and soft tissues) on the plan described by Dr. E. A. Bogue. The description is found in the *Dental Cosmos*, vol. ix. This obturator also consists of two parts, but the joint is rigid with a soft rubber uvula. This part (the soft rubber) doubtless was meant to be the important part of this appliance, but in my experience proved very objectionable on account of becoming very offensive in a short time. Later I substituted

---

[1] To whom the credit of the connecting hinge in obturators with a hard rubber velum (or "chestnut," so called in the *American System of Dentistry*) belongs I have no means of knowing, but there is a description of a hinge and bulb given by George Parkinson, M.R.C.S., of West London Hospital. See *Dental Cosmos*, vol. viii., page 449.

The hollow rubber bulb (or velum) and spring, as described in the *American System of Dentistry*, by Dr. Baker, proved unsatisfactory in my hands, either with or without a spring. With the spring there was an unpleasant grating that annoyed the patient, and without the velum was too light. The hollow rubber bulb, on account of its lightness, might answer a good purpose for children when first wearing an obturator in place of the perishable and offensive soft rubber.

hard rubber, which proved more satisfactory than the soft, which practically made it a rigid appliance.

So far as I know, the obturator is still worn, but in point of comfort and use to the wearer it bears no comparison with the duplex connected with a hinge.

In making this instrument with a hinge I have tried to combine what seemed to me good common sense principles.

While a good cast of all the parts of the mouth with the hard and soft clefts is desirable, it is not essential, as it can only be used as a general outline, not as the means to gain the end.

My method to take an impression is first to take base plate gutta-percha and soften in hot water, and mould to suit the mouth; bend a fairly strong wire and attach to the tray for a handle. This done, fill the tray with softened beeswax and take an impression of all the parts as near as can be taken with this material. This secured, trim the wax wherever it rests against the soft parts, at the same time shaping it so that it will retain the plaster in carrying it to the parts in the mouth.

A few words about plaster and how to use it. The plaster should be about medium in its setting qualities. Fill a mixing-cup about two-thirds full of water, and add small quantities of plaster, adding and stirring until it is about the consistence of thin cream. Keep agitating until the plaster commences to thicken, which may take five or more minutes, if too much plaster has not been added. The least quantity that will do the work will be the most satisfactory. While this method will take more time, the plaster will not be so strong, and will fracture more easily, hurting the patient less.

Before your plaster is ready have the patient in the chair, and as soon as the plaster is in the tray draw the head back with the mouth well opened, put your filled tray in good position in the mouth, and then draw the patient's head forward so as to prevent strangulation, and press the tray with its contents to all the parts as quickly and as gently as you can.

When the plaster left in the mixing-cup will break with a clean fracture, it is an indication to remove the impression, which can only be done in parts, but can be accomplished easily if care has been taken in mixing the plaster and in shaping the wax impression. When all the pieces are removed and placed on the tray, there will still remain some carving in places, and additions to make in others.

To get a perfect impression of the soft parts in their various positions is impossible. Any one that has observed the motions of the cleft tissue knows that the parts are seldom at rest.

Now varnish the impression, oil slightly, invert, and embed the upper half in plaster. When set, trim, varnish, oil, and build up one side with plaster. When set, shape the edges, varnish, and build up the remaining half. After the plaster is well set, if the sections have been properly taken, there will be no trouble in separating them. This done, replace the section of the cast and put away for future reference.

The next important part is to make the front part of the obturator, to which whatever teeth that are needed must be added, and at least one tooth, preferably a molar, on either side of the mouth should be clasped with a good substantial gold clasp attached to the plate.

Before taking the impression for the plate it will be necessary to close the fissure beyond the hard palate with softened wax, so formed that it can be easily removed without injury to the walls of the cleft. The margins of the cleft must, however, be exposed enough to be well defined in the impression, which should be taken with plaster in the same manner as for any other set of teeth.

After a good cast has been secured, proceed as in any other case where a plate with a few teeth and clasps are needed.

The upper side of the plate must be adapted to the margins of the cleft, and also a place provided for the anterior end of the hinge, which may at the same time be attached to the plate. The plate must not extend beyond the hard palate.

But there still remains the most difficult as well as the most important part to do, that of making, attaching, and rightly adjusting the hard rubber to take the place of the lost soft palate.

Before reaching this point, a good, strong, gold hinge should be secured, made of eighteen-carat plate; one thirty-second of an inch thick would not be objectionable.

The width of the cleft must be the guide for the width of the hinge.

The anterior end of the hinge need not be over three-eighths to one-half of an inch in length, and set forward from five-eighths to seven-eighths of an inch from the rear margin of the plate, and should extend from three-eighths to one-half of an inch past the posterior margin of the same, on which to attach the hard rubber bulb. It is important that the joint of the hinge has lateral motion, so as to allow the hard rubber substitute to move from side to side. The object of having the joint of the hinge so far forward is to prevent the rubber bulb from raising at too much of an angle.

After the hinge is connected, form a wax bulb wide enough to fill the space above the cleft, somewhat oblong, rounding edges nearly flat on the nasal side, with a widening and depression towards the posterior, so as to give the lower side a partial arch, rounding towards the sides to allow the cleft tissue to play underneath on either side. Place the plate and the bulb attached in the mouth. Mark the wax wherever you find unequal pressure. Remove the plate and bulb and carve the wax where marked. Again replace and follow these directions until the plate seems comfortable, being careful not to get the wax bulb too small. This done, disconnect the hinge and duplicate the wax bulb in hard rubber.

Polish the rubber, connect the appliance, and place in the mouth, let the patient wear it until points of uneven pressure will make the parts tender. Mark the bulb at the tender points, remove the case, and carve the rubber at the places marked, and again polish, replace, and continue this course until the obturator is comfortable. At no time allow the parts to become chafed.

A lady for whom I made an appliance about six years ago on the plan as here described is as comfortable as it is possible to be under like circumstances. She can enunciate and articulate distinctly with ease, either in conversation or reading aloud.

But for any person to promise, or to state, that a person under such conditions could be enabled by means of *any* appliance to speak or read without betraying the defect to an experienced ear, would simply be an *exaggeration* and a *deception.*

Notwithstanding, when such unfortunates can be helped so as to be comfortable and be enabled to converse with ease and comfort to themselves and pleasure to their friends, we can lay claims to advancement.

There is another matter of no less importance than that of speech in this connection, that is the ability to take food without the necessity of leaving the table to remove particles collecting above the appliance to prevent it (the food) from passing out through the nostrils, as was the case with an obturator formerly worn by my patient, made of one solid piece of hard rubber.

Another difficulty no less unpleasant has been overcome with the duplex obturator she is *now* wearing.

